# Biomechanics

**DOI:** 10.1155/2013/271543

**Published:** 2013-09-02

**Authors:** José M. Vilar, Francisco Miró, Miguel A. Rivero, Giuseppe Spinella

**Affiliations:** ^1^Department of Animal Pathology, Faculty of Veterinary Medicine, University of Las Palmas de Gran Canaria, Trasmontaña S/N, Arucas, 35413 Las Palmas, Spain; ^2^Department of Compared Anatomy and Pathology, Veterinary Faculty, University of Córdoba, Campus de Rabanales, Carretera Madrid-Cádiz Km 396 A, 14071 Córdoba, Spain; ^3^Department of Health Sciences, University Magna Graecia of Catanzaro, Germaneto, 88100 Catanzaro, Italy

Gait evaluation (GA) (kinetic and kinematics) has been generally performed by biomechanics analysis that gather different techniques involved in clinical/experimental human and veterinary studies. Several disciplines in medicine have involved the biomechanics studies for clinical and rehabilitative purposes. In sports, the biomechanics research aimed in ability to establish an understanding of causal mechanisms of movements and how they should be modified in order to achieve better performance. In sports, the role of biomechanics can be categorised into two broad areas: (a) improvement of athletic performance and (b) reducing the injury probability. Recently, the sport biomechanical research has been applied in athletes with a disability, aiming to facilitate improvements in Paralympics athletic performance through applied research and consultancy. In veterinary sciences, the studies are undertaken to characterize and establish the kinematic standards of racing dogs and horses, mainly. Biomechanical study for collecting data of the animal's locomotion has been conducted using different techniques. Some of them, such as skin markers and treadmills, routinely assess the performance status of the animal, but most frequent and minimally invasive methods are videographic and accelerometric techniques. Moreover, gait analysis has been increasingly used in clinical studies to objectively evaluate outcome of medical or surgical treatments in several orthopedic diseases. As other techniques, both kinematic and kinetic gait analyses are extensively used in dogs, due to the usefulness to perform these techniques on animal models in order to extrapolate results for humans. Gait analysis has been involved in pain control of inflammatory status. Force platform has been consistently used as an accurate, objective method to document the efficacy of anti-inflammatory medications such as for the treatment of osteoarthritis (OA). Kinetic analysis of treated subjects is usually performed mounting a force plate in the ground where a dedicated software acquires ground reaction forces (GRF) values in real time. More recently, pressure platforms are being included in GA, since these devices are capable of acquiring pressure distribution data within the limb. Parameters usually acquired are peak vertical forces (PVF) and vertical and horizontal impulses. Moreover, the introduction of inverse dynamics to GA allowed to add new parameters, such as angle, moment, power, and total support moment (TSM), useful for evaluating the joint motion. Moreover, mesenchymal stem cells are gaining an important part in the therapeutic arsenal of multiple pathologies. Locomotor system, especially in degenerative joint diseases, target one of their main indications. Biomechanic gait analysis is being progressively incorporated as a tool to evaluate its efficacy, alone or associated with rich platelet plasma (RPP). Surgical recovery has been consistently evaluated by biomechanic devices in human and veterinary medicine. Cranial cruciate ligament rupture (CCLR) is one of the most important joint diseases both in humans and dogs, based on its prevalence and amount of proposed techniques. Kinematics has also contributed to deep in the knowledge of CCLR joint dynamics, contributing to better understand how CCLR can affect functionality of coxofemoral and tarsal joints, as well as the tibiofemoral joint. This alteration in both hip and tarsal joints could be attributed to specific compensatory modifications. Moreover, hip joint is mostly involved joint in different pathological conditions, due to its anatomic and functional complexity. Coxarthrosis affects both young and ancient subjects, although it is almost directly related to age (70% beyond 65 years). A similar involvement also can be observed in small animals. Multifactorial etiology often occurs, but dysplasia is recognized as one of the most important because it causes joint instability, in human and canine species. 

Lastly, physical therapy has been widely recognised as supporting activity in human beings to return to normal or preinjury function and to prevent age-related deterioration. Electromyographic analysis supplies information on the integral function of the motor system. Kinesiological electromyography can be described as being the study of the neuromuscular activation of muscles within postural tasks, functional movements, work conditions, and treatment/training regimes. Kinesiological EMG can be carried out either by means of surface electrodes or by needle electrodes. Surface electrodes are fixed over the skin of specific muscles, and surface electromyography (sEMG) recordings are the amount of signals from the target muscle and nearby ones. sEMG could be useful in giving valuable information in locomotion of humans and in different domestic quadrupeds such as equines, dogs and cats. Moreover, for humans and, also, for dogs, and horses, treadmill is the most popular fitness and training equipment. Several electromyographic studies in pets have focused on the analysis of muscle activities related to vertebral column and hindlimb movements. The epaxial muscles are assumed to be the primary force for vertebral column movements, extending the back and counteracting the sagittal rebound of the trunk during trotting. To prevent muscle injuries, it is essential to know physiological muscular activity during training or treatment regimes, as well as their changes due to muscular fatigue. Fatigue is the failure to maintain the required or expected force from a muscle following a constant or repeated activity. As it has been stated for human beings, running over inclines or declines can alter muscle function for animals. For quadrupeds, uphill and downhill walking involves postural adjustments of the head, trunk, and limbs. A wide number of rehabilitation exercises are focused on rehabilitation programmes for the spine and the hip joint. Some authors have carried out analyses of electromyographic muscle activity during rehabilitation exercises from these programmes. Isometric and dynamic strengthening exercises are commonly recommended for trunk muscle rehabilitation. Comfort et al. (2011) compared the electromyographic activity of the trunk muscles during both types of exercises. Compared with studies in humans, fewer electromyographic analyses during commonly prescribed therapeutic exercises are available in animals. In horses, the back movements both at walk and at trot have been measured to provide information about the stabilisation forces of the spinal column.

In conclusion, biomechanical evaluation and GA in general will be the cornerstone of scientific success for objective, reliable, and easy tool for clinicians, researchers, and sport field and rehabilitation therapists. Further researches, while continuing to investigate movement in the previously designated categories, must also show how changes in technique and/or equipment design improve performance or health. In this sense, virtual simulation softwares, using a validated computer model, will be able to carry out “experiments” under controlled conditions, with realistic data. 

*José M. Vilar* *Francisco Miró* *Miguel A. Rivero* *Giuseppe Spinella*

